# The impact of significant weight loss following bariatric surgery on the differential expression of phosphopeptides in human seminal fluid

**DOI:** 10.3389/fendo.2026.1907857

**Published:** 2026-10-07

**Authors:** Sally F. Vitez, Odei Barreñada, Kathleen Hwang, Miguel Angel Brieño-Enríquez

**Affiliations:** 1 Department of Obstetrics, Gynecology and Reproductive Science, Division of Reproductive Endocrinology and Infertility, University of Pittsburgh School of Medicine, Pittsburgh, PA, United States; 2 Department of OBGYN and Reproductive Sciences, Magee-Womens Research Institute, University of Pittsburgh, Pittsburgh, PA, United States; 3 Department of Urology, Perelman School of Medicine, University of Pennsylvania, Philadelphia, PA, United States

**Keywords:** bariatric surgery, human, phosphopeptides, protein, seminal fluid

## Abstract

During the last few decades, the global population has shown increased obesity rates. Obesity has been linked to declines in male reproductive capacity, suggesting a causal connection. However, standard fertility tests, such as semen analysis, often fail to predict reproductive success accurately because they do not assess molecular and functional sperm qualities. To address this, this pilot study explores the role of post-translational modifications, such as phosphorylation, as potential fertility markers. We studied six morbidly obese men scheduled for bariatric surgery; all of them were monitored before surgery and then 3 and 6 months afterward. Recruited patients showed statistically significant weight loss and improved hormonal profiles, including higher testosterone and SHBG levels; however, standard semen parameters changed little. Using Mass spectrometry, we evaluated the sperm proteome and phosphoproteome from control patients and patients before and 3 and 6 months after bariatric surgery. Sperm proteomics showed no differences across collection times; however, phosphoproteomics of seminal fluid revealed that 7 phosphopeptides differed significantly across collection times. These post-translational modifications were observed in proteins such as semenogelin-1, semenogelin-2, mucin-6, and AKAP-3 and AKAP-4. Interestingly, these proteins are critical in sperm capacitation, a vital process for fertilization, suggesting a potential molecular mechanism for the reduced reproductive function in obese patients. These results suggest that seminal fluid, rather than sperm alone, may be crucial in mediating changes in fertility associated with obesity and weight loss. Although our results come from a small patient population, the findings highlight the shortcomings of conventional semen analysis and support the potential use of proteomic biomarkers to better understand and assess male fertility amid obesity and metabolic changes.

## Introduction

Over the past 30 years, the rate of obesity has increased in most countries and regions around the world (1). The increasingly sedentary lifestyle and unfavorable diets that are common in Westernized countries increase the risk of diabetes, cancer, and cardiovascular disease in burgeoning obese populations. It is projected that by 2030, 86.3% of all adults in the United States will be overweight or obese, and 51.1% will be obese (2). Interestingly, epidemiological studies have reported declining trends in semen quality and male reproductive potential over the past few decades (3–5). The parallel trends in obesity and declining semen quality suggest a possible cause-and-effect relationship. Infertility constitutes a significant global issue; the CDC estimates 12% or 1 in 8 couples have difficulty getting pregnant (6). It is established that male factor solely accounts for 20% of couples seeking fertility treatment; however, it is probable that it is involved in conjunction with female factors in 30-50% of cases. There is a growing body of evidence supporting that obesity adversely affects spermatogenesis and sperm quality and is associated with a reduction in male reproductive potential (6–13). Obese men are considerably less likely to conceive naturally within a 12-month period of attempting to do so (14). This adverse effect of obesity on fertility and sperm health is thought to operate through mechanisms that modify hormonal profiles in men via the hypothalamic-pituitary-testicular axis (14–17), as well as through increased aromatase activity resulting from elevated adipocyte levels (15–18). However, serum hormones and semen parameters are unreliable measures of reproductive fitness (19). The semen analysis provides limited information and is insufficient to accurately predict male fertility potential (20). Specific limitations of the semen analysis include its inability to evaluate sperm competence to undergo capacitation and to assess the presence of surface proteins required for zona pellucida binding, both of which are required for fertilization. Published prospective studies indicated that the majority of men who lost weight showed no significant changes in semen parameters (semen volume, sperm count, etc.) across time points ranging from 3 to 24 months. The traditional measures of sperm quality, such as motility, count, and semen volume, are poor indicators of fertility status and are susceptible to both intra- and inter-individual variability. The inherent limitations of these traditional measures underscore the need to develop molecular biomarkers of sperm function as a more sensitive indicator. Therefore, it is imperative to conduct a more comprehensive investigation into the fundamental mechanisms at the subcellular level, beyond semen analysis, to precisely evaluate the strong impact of obesity on male reproductive potential.

Proteomics has become a highly prominent area of research over the past decade with many studies examining the human sperm proteome (21–27). As mature sperm and the surrounding seminal fluid proteins can be easily purified from semen, they are ideal candidates for proteomic analysis. Previous publications showed that variations in sperm proteomes occur in clinical conditions, including varicoceles and being overweight. The sperm proteome from men with BMI <25 and >30 showed changes in protein abundance in the obese compared to the control sample (23); obese men had decreased abundance of proteins that play major roles in transcription and translation (EIF3F, EIF4A2). This suggests that obesity could significantly affect gene expression during spermatogenesis, thereby affecting men’s fecundity. However, limited knowledge exists regarding the impact of obesity on the proteome of seminal fluid (28–30) or on the post-translational modifications, such as phosphorylation. Phosphorylation is a reversible post-translational modification that regulates protein function, subcellular localization, and protein degradation. Analysis of protein phosphorylation using mass spectrometry (Phosphoproteomics) can provide a more robust, in-depth view of the cell signaling network and further elucidate molecular biomarkers of disease (31). The true impact of obesity and specifically the impact of weight loss on male fertility within the obese population remains unknown. Identifying and cataloging the sperm and seminal fluid proteomes provides insight into cellular pathways and protein-protein interactions associated with sperm’s fertilizing ability. Herein, we examine and evaluate the impact of obesity and subsequent weight loss on the human sperm and seminal fluid proteome and phosphoproteome.

## Material and methods

### Study subjects

We prospectively recruited patients through the University of Pittsburgh Medical Center (UPMC) Bariatric Surgery Clinic. This pilot study was approved by the Institutional Review Board of the University of Pittsburgh Medical School (IRB: STUDY21070024). Based on previous publications analyzing the sperm proteome, this pilot study evaluated 6 patients and 6 controls. Study subjects were men aged 20–65 years with a BMI >40 who were planning to undergo bariatric surgery and had a normal testicular exam. Before surgery, the Medical Endocrinology service and a certified dietitian evaluated patients. Men were excluded if they were active smokers, had a history of testicular injury, cancer, or surgery, or had a history of infertility. Bariatric surgery (gastric bypass) was performed at a single academic institution by a single surgeon (AB). Exposure to nicotine was evaluated before the surgery. There is no history of use of special medications that can affect protein phosphorylation before or after surgery. No special medications were used before or after the surgery. Control patients were men aged 20–65 years, with BMI below 25, who planned to undergo semen analysis (SA) for clinical evaluation at the UPMC Magee-Womens Center for Fertility and Reproductive Endocrinology (CFRE), and who planned to undergo vasectomy with proven fertility and normal weight (BMI<25). Exclusion criteria for control patients were a history of male factor infertility, sperm density <10 million or azoospermia, a history of vasectomy or planned cryopreservation for an assisted reproductive technology procedure (testicular biopsies), active smoking, a history of testicular injury, cancer, or surgery. Following standard care for bariatric surgery patients, we evaluated them at three time points: preoperatively (time point 0, within 7 days before surgery), 3 months postoperatively, and 6 months postoperatively (± 7 days). At each visit, we measured height, weight, and abdominal circumference, and collected blood and semen samples. Control patients were sampled once. We compared controls and patients in pairs at baseline; we analyzed changes over time using repeated measures so each patient could be compared to themselves across visits. We performed statistical analyses using GraphPad Prism 11.0.2 (100). We used repeated-measures ANOVA for the morphometric. We compared controls and patients using a paired t-test. We tested age and other variables as potential confounders.

### Blood and semen processing and analysis

For both patients and controls blood samples were collected through standard phlebotomy at a clinical academic center (UPMC). Semen samples were collected through masturbation after 48–72 hours of abstinence. A clinical andrologist processed the samples and performed a standard WHO semen analysis (SA). The remaining samples were centrifuged at 600g for 15 minutes. Seminal removed the seminal plasma and cryopreserved it in liquid nitrogen. The sperm pellet was resuspended in 0.2mL of HEPES media and cryopreserved in liquid nitrogen upon processing and analysis for mass spectrometry. Freeze-thaw cycles were avoided. Statistical analysis was performed using GraphPad Prism 11.0.2 (100). We used repeated-measures ANOVA for the hormonal and sperm analyses. We compared controls and patients using a paired t-test.

### Western blot

Before cryopreservation, a small fraction of the seminal fluid and sperm pellet was used to validate the separation techniques for sperm and seminal fluid by Western blot. Sample protein concentration was measured using the Pierce BCA protein assay to determine the protein concentration of all samples. For all samples, 10 micrograms of protein from each sample was separated by electrophoresis on a 4-12% gel and transferred to a nitrocellulose membrane (all of them from BioRad). After transferring, the membrane was blocked with 3% bovine serum albumin at 4 °C overnight. The membrane was then washed and incubated with the primary antibody at 4 °C overnight. The primary antibodies were directed against the following: Sperm protamine P2 protein (PRM2) (Proteintech - 14500-1-AP; 1:1000), ADAM2 antibody (Thermo Scientific - PA5-87699; 1:1000). The membrane was then washed and incubated with the secondary horseradish peroxidase (HRP) - conjugated anti-rabbit (Jackson ImmunoResearch; 111-036-045; 1:1000) antibody at room temperature for 2 hours. Signal detection was performed using SuperSignalTM West Pico PLUS Chemiluminescent substrate kit (Thermo Scientific #34580) with the ChemiDOCTM MP imaging System (BioRad). The nitrocellulose membrane was then stripped and re-incubated with (HRP)- conjugated beta-actin (anti-rabbit; Thermo Scientific - MA5-32540) as a loading control, with imaging performed as outlined above.

### Sample preparation, protein digestion, and mass tag labeling

All samples (patients and controls) were processed and analyzed by the Mass Spectrometry Proteomics Core at the University of Pittsburgh. To avoid technical variations, all samples were collected and stored under the same conditions, controlling unnecessary freeze-thaw cycles. Samples were randomized to ensure sample processing and run order and included pooled quality control (QC). For all the processes, samples were processed at the same facility, at the same time, and by the same user. Patient and control samples were sonicated, and total protein was quantified using the BCA assay. A global internal standard (GIS) was prepared by mixing a small aliquot from each sample, and the GIS was included in each TMT plex as described below. Fifty micrograms of protein from each subject and GIS were digested with trypsin at a 1:20 ratio using S-trap micro columns (Protifi). Samples were isobarically labeled by incubation with TMTpro reagent at a 1:4 peptide: reagent ratio for 1 h, followed by quenching with 5% hydroxylamine. TMT plexes were pooled, with each plex receiving two or three GIS. Samples were then desalted using Pierce Peptide Desalting Spin Columns (ThermoFisher Scientific) and underwent phosphopeptide enrichment on an Agilent AssayMAP with Fe(III)-NTA cartridges. Samples were then fractionated using Pierce High pH Reversed-Phase Peptide Spin Columns (ThermoFisher Scientific). Peptides were eluted into 5 fractions using acetonitrile at 10%, 12.5%, 15%, 17.5%, and 50%, dried, and resuspended in 3% acetonitrile and 0.1% formic acid.

### Mass spectrometry

Mass spectrometry analysis was conducted on a Thermo Fisher QE-HFX coupled to an Easy-nLC 1200. Approximately 1 ug of each sample was loaded onto an EASY-Spray PepMap RSLC C18 column (2 um, 100A, 75um x 50 cm) and eluted at 300 nl/min over a 180-minute gradient. MS1 spectra were collected at 120,000 resolutions with a full-scan range of 350–1500 m/z, a maximum injection time of 50ms, and automatic gain control (AGC). The precursor selection window was set to 0.7 m/z, and fragmentation was performed at a normalized collision energy of 34%. MS2 were collected with a resolution of 60,000, a maximum injection time of 350ms, AGC set to 5e4, and dynamic exclusion time set to 90s. Raw data files were analyzed with Proteome Discoverer (ThermoFisher Scientific) and searched against the human SwissProt database using Sequest HT (missed cleavages = 3; minimum peptide length = 6; precursor mass tolerance = 10 ppm; dynamic modifications: Phosphorylation/+ 79.966, Oxidation/+ 15.995 Da, Acetyl/+ 42.011 Da, Met-loss/-131.040 Da, Met-loss+Acetyl/-83.030 Da; static modifications: TMTpro/+304.207 Da Carbamidomethyl/+57.021 Da). Peptide spectral matches were filtered using Percolator with a dCN = 0.05 and FDR = 1%. Statistical analysis was performed in R using MSstatsTMT (v2.18.0). Each TMT experiment comprised two 11-plexes of biological channels and three pooled global internal standard (GIS) channels used as between-run normalization references. Patients were linked across conditions using a shared biological replicate identifier to enable repeated-measures modeling. Peptide-level data underwent global median normalization, AFT-based imputation of censored missing values, and GIS-based normalization between runs before protein summarization. Differential abundance was tested with a linear mixed-effects model with empirical Bayes moderation across six pre-specified contrasts, with Benjamini–Hochberg correction; adjusted p < 0.05 was considered statistically significant. Functional enrichment was performed with clusterProfiler (4.18.4) against the Gene Ontology and KEGG databases, using the full detected proteome as the background (adjusted p < 0.05). Results were visualized with ggplot2.

## Results

Six morbidly obese men (mean BMI 51.2) and the corresponding controls (mean BMI 23.7) were enrolled, with mean ages of 42.3 years and 37.1 years, respectively. All 6 obese men and 6 controls remained enrolled throughout the study. All 6 patients underwent gastric bypass surgery without complication, performed at the same hospital and by the same minimally invasive surgeon. We measured weight before surgery (T0), at 3 months (T3MO), and at 6 months (T6MO). All participants had significant weight loss, with average reductions of 21% at T3MO (129.4 ± 22.22) and 24% at T6MO (117.2 ± 19.51) (**Table 1**, **Figure 1A**). We analyzed body mass index (BMI) at different time points. The average BMI before surgery was 51.22. Patients at T3MO and T6MO showed a statistically significant reduction in BMI (Table, **Figure 1B**). Similar to what was observed with weight and BMI, waist circumference measurements were obtained at preoperative (mean 149.9 ± 11.71), 3 (125.5 ± 16.05), and 6 (102.9 ± 18.41) months postoperative (**Table 1**; **Figure 1C**). Weight loss is associated with improved metabolism, and to assess whether weight loss was also associated with changes in pituitary hormone levels, we evaluated luteinizing hormone (LH), follicle-stimulating hormone (FSH), and estrogen levels (**Table 1**). Consistent with previous publications, we observed no statistically significant differences across time points (**Table 1**, **Figures 1D–F**). We also evaluated the total testosterone, free testosterone, bioactive testosterone, and sex hormone-binding proteins (**Table 1**, **Figures 1G–I**). Total testosterone increased significantly at T3MO (407.7 ± 165.8 ng/dL) and T6MO (511.5 ± 182.7 ng/dL). There was also a significant increase in sex hormone-binding globulin (SHBG; T3MO 40.17 ± 7.80; T6MO 42.67 ± 10.60), with an increase in the testosterone-to-estrogen (T to E) ratio (**Table 1**, **Figures 1G–I**). No differences were observed in bioavailable testosterone and free testosterone (**Table 1**; **Figure 1H, I**). We analyzed all patients’ semen according to WHO standard semen analysis. No differences were observed in semen volume (T0 vs T3MO, p=0.8977; T0 vs T6MO, p=0.9044; T3MO vs T6MO, p= 0.6652), density (T0 vs T3MO, p=0.7209; T0 vs T6MO, p=0.9308; T3MO vs T6MO, p= 0.9088), motility (T0 vs T3MO, p=0.9258; T0 vs T6MO, p=0.7138; T3MO vs T6MO, p= 0.9094), and morphology (**Table 2**). Interestingly, we observed a trend toward decreased progressive motility (T0 vs T3MO, p=0.9117; T0 vs T6MO, p=0.8011; T3MO vs T6MO, p= 0.3228) (**Table 2**).

**Table 1:** Extended clinical information for controls and patients across timepoints.

	CONTROL		T0					T3MO					T6MO				
	Mean	SD	Mean	SD	25^th^	50^th^	75^th^	Mean	SD	25th	50^th^	75^th^	Mean	SD	25th	50th	75th
Weight (kg)	73.55	5.1	166.7	28.7	148.6	166.4	184.6	129.4	22.2	116.2	129.8	140.4	117.2	19.5	108.8	115.3	127.8
BMI (kg/m2)	23.78	1.9	51.2	6.9	45.8	51.9	56.9	39.7	5.4	35.7	39.4	44.8	36.0	4.8	32.3	34.9	40.9
Waist Circumference (cm)	82.2	3.4	149.0	12.7	141.2	150.6	157.4	123.5	14.6	105.4	119.3	136.2	119.3	13.7	108.4	123.1	130.3
LH	6.5	1.8	7.0	3.1	4.9	5.7	9.2	7.6	2.1	5.8	7.8	10.2	6.2	2.8	4.1	5.5	7.8
FSH	6.2	2.1	5.3	3.7	3.0	4.6	6.9	4.7	2.2	3.2	4.2	6.0	5.3	2.5	3.7	4.7	7.6
Estrogen (E2)	25.2	6.4	47.3	15.5	35.0	43.0	60.3	41.2	13.2	28.9	41.3	44.6	44.1	14.1	29.6	47.6	54.7
Free Testosterone	65.1	14.6	39.4	15.6	28.3	35.7	48.7	46.7	18.7	29.5	41.7	65.7	58.6	26.1	43.6	49.8	76.8
Total Testosterone	430.2	136.1	242.7	81.8	176.8	232.5	285.0	407.6	165.8	273.0	336.5	595.2	511.5	182.7	356.2	453.5	728.7
Bioavailable Testosterone	106.5	34.1	78.0	37.0	55.5	67.9	94.1	91.4	42.0	56.5	79.3	128.7	113.8	56.7	79.92	96.9	143.9
Albumin	4.0	0.4	4.2	0.3	4.0	4.3	4.4	4.0	0.3	3.8	3.9	4.4	4.2	0.30	3.9	4.2	4.4
SHBG	44.2	26.9	24.7	5.0	21.0	24.0	29.0	40.1	7.1	33.5	40.0	47.2	42.7	10.6	32.5	42.5	52.2

**Figure 1:**
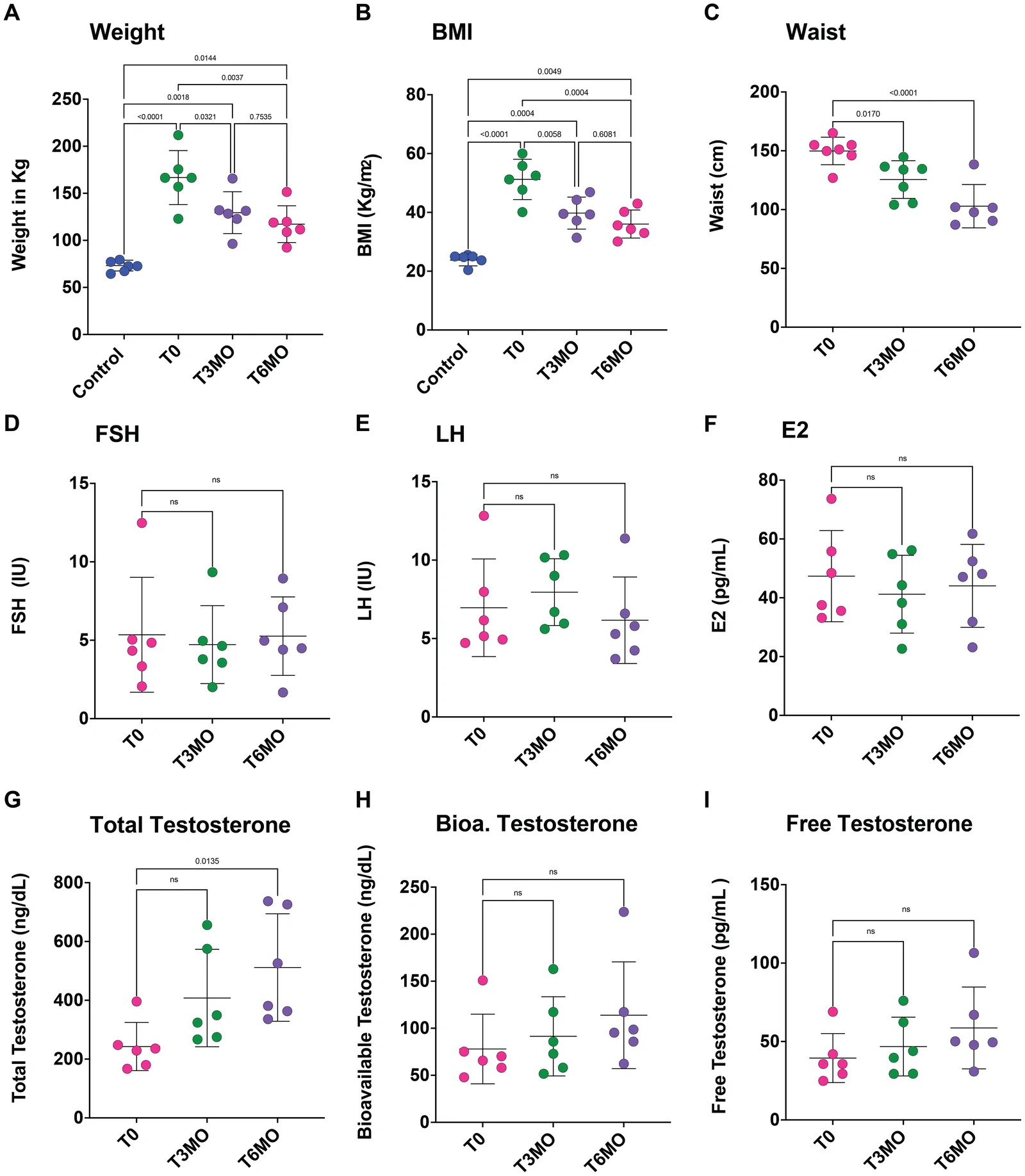
Clinical parameters across patient time points. **(A)** Analysis of weight loss across different time points in Kg (Control; T0 collection before surgery; T3MO collection 3 months after surgery; and six months after surgery, T6MO). **(B)** Evaluation of BMI across time points; **(C)** Measurement of waist perimeter in cm. **(D–I)** Detection of FSH, LH, E2, Total testosterone, bioavailable testosterone, and free testosterone across different time points (Control; T0 collection before surgery; T3MO collection 3 months after surgery; and six months after surgery, T6MO). Statistical analysis was performed using GraphPad Prism 11.0.2 (100). We used repeated-measures ANOVA for the hormonal and sperm analyses. We compared controls and patients using a paired t-test. NS, not statistically significant; p-values are represented in the graph.

**Table 2:** Evaluation of semen using WHO parameters across the timepoints.

	CONTROL		T0		T3MO		T6MO	
	Mean	SD	Mean	SD	Mean	SD	Mean	SD
Semen Volume	2.34	1.23	2.45	1.89	2.02	1.58	2.86	1.50
Density	55.8	11.96	45.07	26.99	29.33	26.02	37.77	47.57
TMC	120.66	37.73	95.12	64.1	56.04	68.23	114.62	66.43
Motility	63.2	15.51	52.08	15.51	47.50	17.35	42.50	23.28
Progressive Motility	80	16.55	56.00	30.21	51.00	20.58	43.17	22.80
Morphology	3.60	1.14	2.16	1.60	1.50	1.51	2.00	2.28

To assess whether weight loss changes the sperm and seminal fluid proteome, we analyzed sperm and seminal fluid purity after fluid separation and confirmed the results by Western blotting (**Supplementary Figure 1**). The sperm proteome did not differ between control patients and obese patients at T0, T3MO, and T6MO (**Figure 2A**; **Supplementary Table 1**-**4**). However, the seminal fluid proteome analysis showed significant differences across all conditions. The number of statistically significant proteins varies across comparisons; however, the greatest changes in the proteome were observed when T0 is compared with T3MO, with 46 proteins (**Figure 2B**; **Supplementary Table 1**-**4**) followed by T3MO vs control with 30 proteins (**Figure 2B**; **Supplementary Table 1**-**4**). Using GO analysis, we evaluated the enriched biological processes (**Figure 2C**); these included reproductive processes, secretion, exocytosis, sperm motility, and acrosome reaction (T3MO vs control) (**Figure 1C**; **Supplementary Table 5**-**6**). Comparison of T0 vs T3MO shows enrichment for flagellated sperm motility, cilium/flagellum-dependent cells, and motility (**Figure 2D**). Following global proteomics, we analyzed the phosphoproteome and identified a total of 618 phosphopeptides in sperm and 270 in seminal fluid. Data filtration removed low-confidence peptides, resulting in 591 sperm phosphopeptides and 192 seminal fluid phosphopeptides. No differentially regulated phosphopeptides were identified in sperm samples over time (**Figure 2D**, **Supplementary Table 1-4**). Seven differentially regulated phosphopeptides were observed across the different comparisons, these posttranslational modifications were observed on A-Kinase Anchoring Protein 3 (AKAP3); A-Kinase Anchoring Protein 4 (AKAP4), Muci-6 (MUC6); Semenogelin-1 (SEMG1), Sperm Acrosome Associated 1 (SPACA1), Serine/Threonine/Tyrosine Interacting Like 1 (STYL1), and Transglutaminase 4 (TGM4) (**Figure 2D**). Only AKAP3 was dysregulated across the different collection times, showing a monotonic pattern with a statistically significant upregulation of phosphorylation at threonine T767 (T0 vs T3MO and T0 vs T6MO), and followed by a partial reversion to a statistically significant downregulation of the same residue, T767, in the comparison of T3MO vs T6MO (**Figure 1D**). Interestingly, AKAP3 also showed upregulation at serine 760 (S760) when we compared T0 vs T6MO. In the case of AKAP4, weight loss induced an upregulation of threonine 491 (T491) (**Figure 2D**). Mucine-6 showed lower levels of phosphorylation of serine 1023 compared to T6MO, followed by hyperphosphorylation of the same residue at T6MO (**Figure 2D**). The next differentially regulated phosphopeptide was identified on the parent protein Semenogelin-1, with reduced phosphorylation at serine 290 and 291. It was upregulated at 6 months compared with 3 months (**Figure 2D**). SPACA1 and SYTL1 showed downregulation of phosphorylation at threonine residues T257 and S392, respectively (T0 vs T6MO) (**Figure 2D**). Also, at 6 months, we observed upregulation of serine 54 (S54) in TGM4.

**Figure 2:**
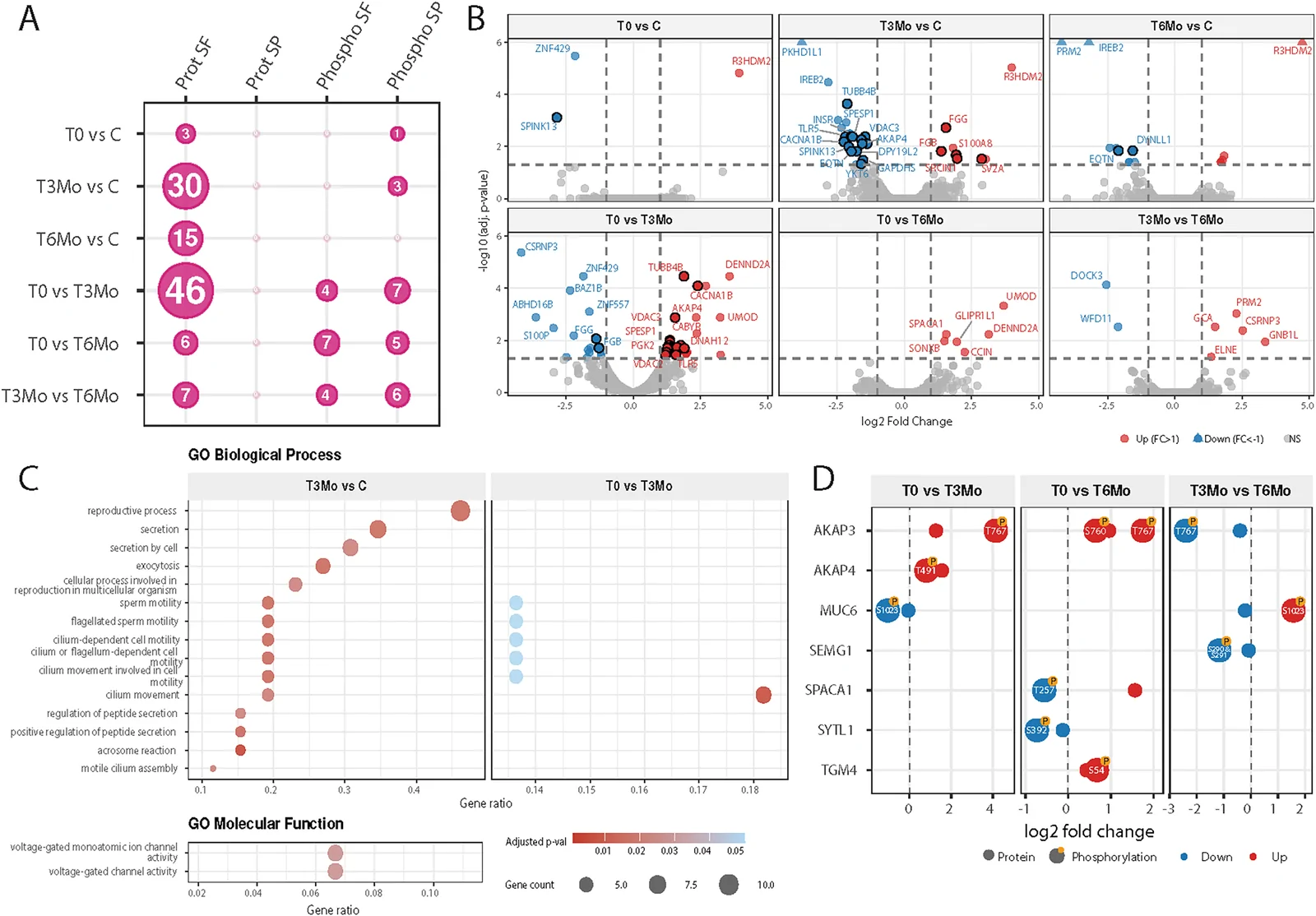
Proteomic analysis of seminal fluid and sperm across patient time points. **(A)** Number of significantly differentially abundant proteins or phosphopeptides per pairwise comparison and dataset (adjusted p < 0.05). **(B)** Volcano plots for all six comparisons in the global proteome seminal fluid dataset (ProtTMT_SF). Each point represents one protein; red, upregulated; blue, downregulated (|log2FC| > 1, adjusted p < 0.05). Proteins contributing to enriched pathways are outlined in black. Labels are shown for a representative subset. **(C)** Gene Ontology enrichment dot plots (Biological Process and Molecular Function) for the ProtTMT_SF dataset. Point size reflects the number of genes annotated to each term; color indicates adjusted p-value. **(D)** Paired comparison of log2 fold changes at the phosphopeptide and total protein levels for proteins significant in both assays within the same comparison. Abbreviations: SF, seminal fluid; SP, sperm; T, timepoint; Mo, months.

## Discussion

Over the last few decades, not only is obesity a global pandemic, but it has also been associated with subfertility and infertility. We evaluated the impact of bariatric surgery on the regulation of sperm and seminal fluid proteomics. In accordance with previous reports, we observed increased free and total testosterone, increased SHBG, an elevated T:E ratio, and no significant differences in semen parameters (32, 33). We did not identify any other impact on the hypothalamic-pituitary-testicular axis with normal levels of LH, FSH, and E2. It has previously been reported that semen analyses are not truly representative of male reproductive potential (34, 35), and given the significant physiological changes associated with rapid weight loss in this cohort of men, we sought to evaluate the impact on fertility using phosphoproteomic analysis. Using highly sensitive, quantitative TMT-based phosphoproteomics, we identified 7 proteins with differentially expressed phosphopeptides in the seminal fluid of obese men undergoing rapid weight loss after bariatric surgery, with AKAP3 being the most robust. We did not identify any differentially expressed phosphopeptides in the sperm over the same time course within this cohort. Although interesting, this is not entirely surprising, as it helps us better understand the impact of seminal fluid on sperm function and male fertility. Sperm cells comprise < 10% of semen volume, with the remainder being seminal fluid (29, 36). Seminal fluid plays a pivotal role in natural fertilization as it is composed of diverse proteins that aid sperm transport, immunomodulation, and capacitation (28, 29, 37). Capacitation is a prerequisite for natural fertilization and consists of the cellular and molecular changes that spermatozoa must undergo to fertilize a mature oocyte. It consists of three main parts, the ability to bind the zona pellucida, the ability to undergo the acrosome reaction, and the acquisition of hypermobility. It is well understood that during capacitation, cell membranes become more permeable through removal of cholesterol. This increase in membrane permeability allows the influx of calcium, which activates cAMP and leads to protein phosphorylation, an absolute requirement for capacitation (38). A-Kinase Anchor Proteins (AKAP) are family scaffolding proteins that function to tether the regulatory subunits of protein kinase A to organelles or cytoskeletal elements allowing tight control of signal transduction cascades (38, 39). Specifically, AKAP-3 and 4 are the most abundant proteins in the sperm fibrous sheath and are actively involved in the acquisition of sperm hypermobility during capacitation (38–44). One prior study by Ficarro et al. identified a phosphopeptide on the AKAP4 protein that was significantly upregulated during sperm capacitation, suggesting it may be involved in regulate AKAP4 function during capacitation (39). Our study highlights upregulation of a unique phosphopeptide within the parent protein AKAP3 and 4, with one serine phosphorylation and three threonine sites. Although the phosphosites are not at the same specific residues, it clearly demonstrates the relevance of AKAP protein phosphorylation. This was observed when comparing the pre-operative and 3-month time points with the 6-month time point. This suggests that up-regulation is induced after weight loss has begun and persists as weight loss continues. While no specific change was identified in standard semen analysis, as previously discussed, semen analysis is insufficient to represent the fertility status of a male partner (34, 35). Thus, further understanding the specific role of phosphopeptide upregulation in AKAP3 and 4, and its downstream effects on capacitation, would help elucidate how significant weight loss affects the sperm’s ability to undergo capacitation and achieve natural fertilization. Prior studies have identified both Semenogelin I (SEMG1) and Semenogelin II (SEMG2) as proteins expressed in the seminal fluid of men with proven fertility (30, 45, 46). The SEMG1 and SEMG2 proteins secreted by the seminal vesicle are basic fibrous proteins that form a coagulum of ejaculated semen (47, 48), yet they are lysed by prostate specific antigen (PSA) typically soon after ejaculation (49–52). Although it is well described that the role of SEMG1 and SEMG2 proteins is to facilitate coagulation, multiple studies highlight other potential protein functions (47, 49, 52). Specifically, Lamirande et al. identified how Semenogelin proteins inhibit human sperm capacitation in a dose-dependent manner (47). They also find that high concentrations of Semenogelin completely immobilize sperm. Given the rapid metabolism of semenogelin by PSA, it is hypothesized that degradation products remain biologically active at low concentrations and help prevent hyperactivation of sperm and early capacitation (47, 50).

Overall, our phosphoproteomic findings suggest that obesity-related metabolic problems are more likely to change protein phosphorylation in seminal plasma than in sperm cells. The pathway from obesity leading to metabolic dysfunction and oxidative stress is strongly supported, as is the pathway from cAMP/PKA to AKAP phosphorylation and then to capacitation (53–56). The idea that obesity causes abnormal phosphorylation of seminal plasma proteins, which then adversely affects sperm signaling, is a reasonable and biologically feasible model, although it still needs to be confirmed through direct experiments. This is biologically plausible because seminal plasma proteins are secreted by active accessory glands, whereas mature sperm exhibit limited capacity for transcription and translation, thereby maintaining a relatively stable phosphoproteome. Among the proteins showing altered phosphorylation, SEMG1 is a key decapacitation factor from the seminal vesicles that helps regulate semen coagulation, sperm coating, and the timing of capacitation (57, 58). If SEMG1 is abnormally phosphorylated, it could change its structure or how it’s processed, which might delay sperm release and hinder bicarbonate-induced activation of the soluble adenylyl cyclase (sAC), which produces cyclic AMP (cAMP), which then activates protein kinase A (PKA) (sAC-cAMP-PKA signaling pathway) (59, 60). This impaired pathway activation could, in turn, reduce phosphorylation and remodeling of AKAP3 and AKAP4, critical scaffold proteins in the fibrous sheath that organize cAMP signaling necessary for sperm hyperactivation and the acrosome reaction. So, these changes in seminal plasma phosphorylation linked to obesity might indirectly affect sperm capacitation, even if there are no visible differences in the basal phosphoproteins of sperm. While the exact effects of the identified SEMG1 phosphorylation sites still require experimental confirmation, this model offers a useful framework linking metabolic problems, accessory gland issues, impaired capacitation, and reduced male fertility. Without a doubt, further research into the specific phosphopeptides identified in our study will help elucidate their roles in capacitation and motility, as well as their impact on male fertility. Changes in phosphorylation following significant, rapid weight loss, particularly in phosphopeptides involved in capacitation, suggest that obesity impairs the proper regulation of capacitation, a mechanism required for natural fertilization. Although all patients underwent the same surgery and, in general, took the same medications, this study cannot distinguish how post-surgery inflammation or dietary changes independent of weight per se may modify the seminal fluid phosphoproteome. Further mechanistic analyses are needed to evaluate the specific role of the regulation of phosphopeptides outlined above.

## Study limitations

Our study used a longitudinal design to at least partially compensate for the small sample size (n=6). We acknowledge that, for human populations, these results are considered exploratory and that larger patient cohorts are needed. Although these initial results are promising, functional validation is needed through prospective studies that follow patients and analyze fertilization capacity and pregnancy outcomes. We acknowledge the lack of mechanistic studies using animal models in which phosphorylation sites are experimentally modified (phosphomimetic and phosphomutant), as well as secondary validation of the phosphoproteomics using custom-made antibodies targeting specific phosphosites.

## Conclusion

Bariatric surgery has a positive effect on total testosterone, free testosterone, bioactive testosterone, and sex hormone-binding proteins. Bariatric surgery has the potential to alter the seminal fluid phosphoproteome. Our results suggest that seminal fluid, rather than sperm alone, may be crucial in mediating fertility changes related to obesity and weight loss. Although larger longitudinal studies in humans and mechanistic studies in animal models need to be performed, our results highlight the shortcomings of conventional semen analysis and advocate for proteomic biomarkers to better understand and assess male fertility amid obesity and metabolic changes. However, larger longitudinal studies and mechanistic analyses are needed. Overall, the findings highlight the shortcomings of conventional semen analysis and advocate for proteomic biomarkers to better understand and assess male fertility amid obesity and metabolic changes.

## Data Availability

The original contributions presented in the study are publicly available. This data can be found in the PRID database, with project accession number PXD081776 and project DOI 10.6019/PXD081776.

## Supplementary material

Supplementary Table 1 Tables of comparison results. One tab per dataset, with all comparisons included. Tables of comparison results. Each dataset is provided in a separate worksheet containing all corresponding pairwise comparisons. An additional fifth worksheet summarizes the statistically significant findings in seminal fluid, highlighting proteins identified as differentially abundant at both the total proteome and phosphoproteome levels.

Supplementary Table 2 Tables of comparison results. One tab per dataset, with all comparisons included. Tables of comparison results. Each dataset is provided in a separate worksheet containing all corresponding pairwise comparisons. An additional fifth worksheet summarizes the statistically significant findings in seminal fluid, highlighting proteins identified as differentially abundant at both the total proteome and phosphoproteome levels.

Supplementary Table 3 Tables of comparison results. One tab per dataset, with all comparisons included. Tables of comparison results. Each dataset is provided in a separate worksheet containing all corresponding pairwise comparisons. An additional fifth worksheet summarizes the statistically significant findings in seminal fluid, highlighting proteins identified as differentially abundant at both the total proteome and phosphoproteome levels.

Supplementary Table 4 Tables of comparison results. One tab per dataset, with all comparisons included. Tables of comparison results. Each dataset is provided in a separate worksheet containing all corresponding pairwise comparisons. An additional fifth worksheet summarizes the statistically significant findings in seminal fluid, highlighting proteins identified as differentially abundant at both the total proteome and phosphoproteome levels.

Supplementary Table 5 Gene pathway enrichment analysis. Summary of enriched pathways detected in each dataset and comparison using the differentially detected peptides.

Supplementary Table 6 Gene pathway enrichment analysis. Summary of enriched pathways detected in each dataset and comparison using the differentially detected peptides.

Supplementary Figure 1 Volcano plots of all comparisons (“T0 vs C”, “T3Mo vs C”, “T6Mo vs C”, “T0 vs T3Mo”, “T0 vs T6Mo”, “T3Mo vs T6Mo”) of the 4 datasets (“ProtTMT_SF”, “ProtTMT_SP”, “PhosphoTMT_SF” and “PhosphoTMT_SP”).

Supplementary Figure 2 Volcano plots of all comparisons (“T0 vs C”, “T3Mo vs C”, “T6Mo vs C”, “T0 vs T3Mo”, “T0 vs T6Mo”, “T3Mo vs T6Mo”) of the 4 datasets (“ProtTMT_SF”, “ProtTMT_SP”, “PhosphoTMT_SF” and “PhosphoTMT_SP”).

Supplementary Figure 3 Volcano plots of all comparisons (“T0 vs C”, “T3Mo vs C”, “T6Mo vs C”, “T0 vs T3Mo”, “T0 vs T6Mo”, “T3Mo vs T6Mo”) of the 4 datasets (“ProtTMT_SF”, “ProtTMT_SP”, “PhosphoTMT_SF” and “PhosphoTMT_SP”).

Supplementary Figure 4 Volcano plots of all comparisons (“T0 vs C”, “T3Mo vs C”, “T6Mo vs C”, “T0 vs T3Mo”, “T0 vs T6Mo”, “T3Mo vs T6Mo”) of the 4 datasets (“ProtTMT_SF”, “ProtTMT_SP”, “PhosphoTMT_SF” and “PhosphoTMT_SP”).
