# Supplementary material for: The impact of significant weight loss following bariatric surgery on the differential expression of phosphopeptides in human seminal fluid
Source: Front Endocrinol (Lausanne). 2026 Oct 7;17:1907857. doi: 10.3389/fendo.2026.1907857 (PMC13684861; doi:10.3389/fendo.2026.1907857)
Supplement: Supplementary file 1 [file DataSheet1.pdf]

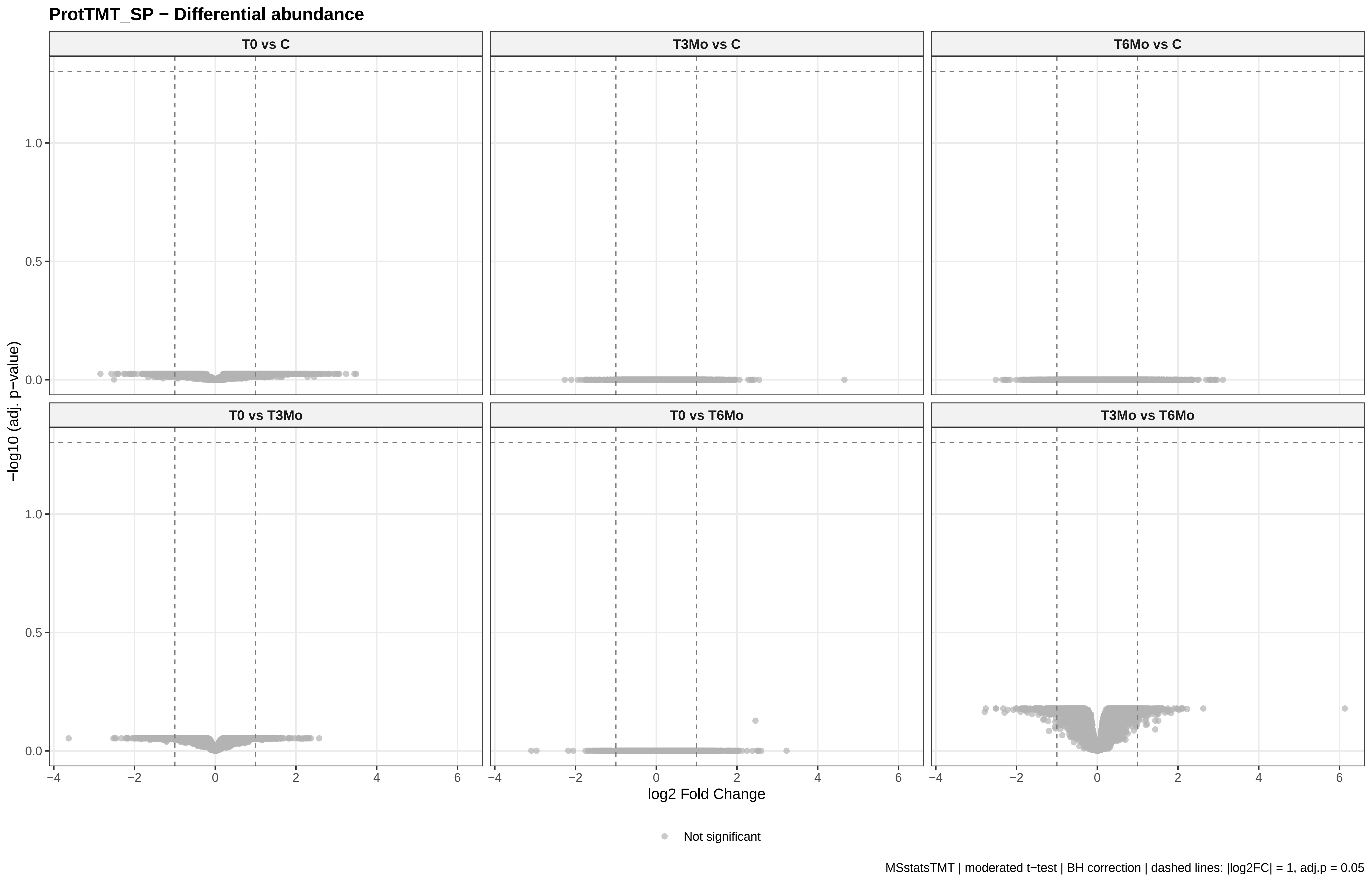
ProtTMT_SP − Differential abundance
T0 vs C
T3Mo vs C
T6Mo vs C
T0 vs T3Mo
T0 vs T6Mo
T3Mo vs T6Mo
−log10 (adj. p−value)
1.0
0.5
0.0
−4
−2
0
2
4
6
log2 Fold Change
Not significant
MSstatsTMT | moderated t−test | BH correction | dashed lines: |log2FC| = 1, adj.p = 0.05
