# Supplementary material for: The impact of significant weight loss following bariatric surgery on the differential expression of phosphopeptides in human seminal fluid
Source: Front Endocrinol (Lausanne). 2026 Oct 7;17:1907857. doi: 10.3389/fendo.2026.1907857 (PMC13684861; doi:10.3389/fendo.2026.1907857)
Supplement: Supplementary file 2 [file DataSheet2.pdf]

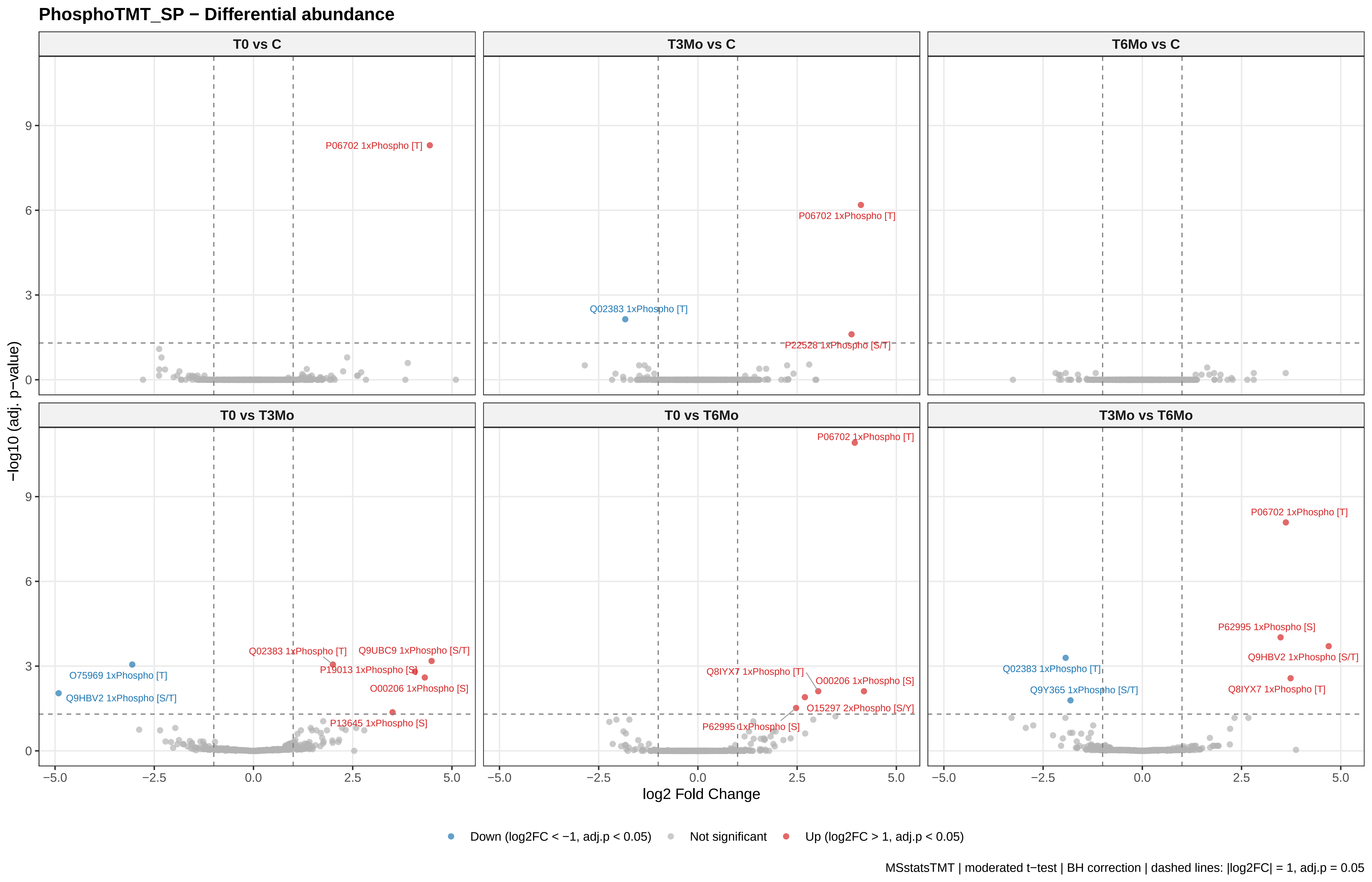

PhosphoTMT_SP − Differential abundance
T0 vs C
T3Mo vs C
T6Mo vs C
T0 vs T3Mo
T0 vs T6Mo
T3Mo vs T6Mo
P06702 1xPhospho [T]
Q02383 1xPhospho [T]
P22528 1xPhospho [S/T]
O75969 1xPhospho [T]
Q9HBV2 1xPhospho [S/T]
P19013 1xPhospho [S]
Q9UBC9 1xPhospho [S/T]
O00206 1xPhospho [S]
P13645 1xPhospho [S]
Q8IYX7 1xPhospho [T]
O15297 2xPhospho [S/Y]
P62995 1xPhospho [S]
Q9Y365 1xPhospho [S/T]
−log10 (adj. p−value)
log2 Fold Change
Down (log2FC < −1, adj.p < 0.05)
Not significant
Up (log2FC > 1, adj.p < 0.05)
MSstatsTMT | moderated t−test | BH correction | dashed lines: |log2FC| = 1, adj.p = 0.05
