# Supplementary material for: The impact of significant weight loss following bariatric surgery on the differential expression of phosphopeptides in human seminal fluid
Source: Front Endocrinol (Lausanne). 2026 Oct 7;17:1907857. doi: 10.3389/fendo.2026.1907857 (PMC13684861; doi:10.3389/fendo.2026.1907857)
Supplement: Supplementary file 3 [file DataSheet3.pdf]

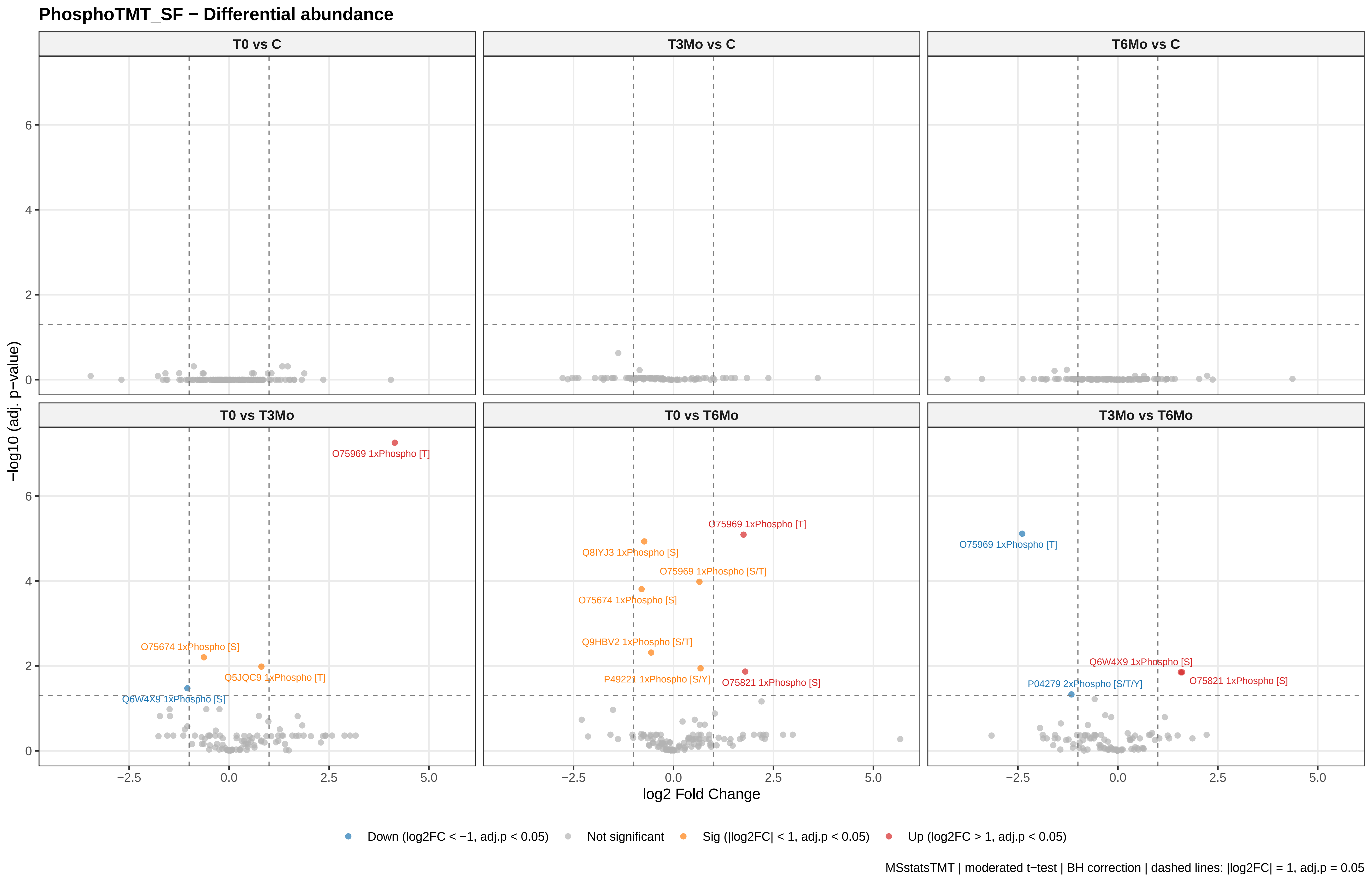

PhosphoTMT_SF − Differential abundance
T0 vs C
T3Mo vs C
T6Mo vs C
T0 vs T3Mo
T0 vs T6Mo
T3Mo vs T6Mo
−log10 (adj. p−value)
log2 Fold Change
O75969 1xPhospho [T]
O75674 1xPhospho [S]
Q5JQC9 1xPhospho [T]
Q6W4X9 1xPhospho [S]
O75969 1xPhospho [T]
Q8IYJ3 1xPhospho [S]
O75969 1xPhospho [S/T]
O75674 1xPhospho [S]
Q9HBV2 1xPhospho [S/T]
P49221 1xPhospho [S/Y]
O75821 1xPhospho [S]
O75969 1xPhospho [T]
Q6W4X9 1xPhospho [S]
P04279 2xPhospho [S/T/Y]
O75821 1xPhospho [S]
Down (log2FC < −1, adj.p < 0.05)
Not significant
Sig (|log2FC| < 1, adj.p < 0.05)
Up (log2FC > 1, adj.p < 0.05)
MSstatsTMT | moderated t−test | BH correction | dashed lines: |log2FC| = 1, adj.p = 0.05
