# Supplementary material for: The impact of significant weight loss following bariatric surgery on the differential expression of phosphopeptides in human seminal fluid
Source: Front Endocrinol (Lausanne). 2026 Oct 7;17:1907857. doi: 10.3389/fendo.2026.1907857 (PMC13684861; doi:10.3389/fendo.2026.1907857)
Supplement: Supplementary file 4 [file DataSheet4.pdf]

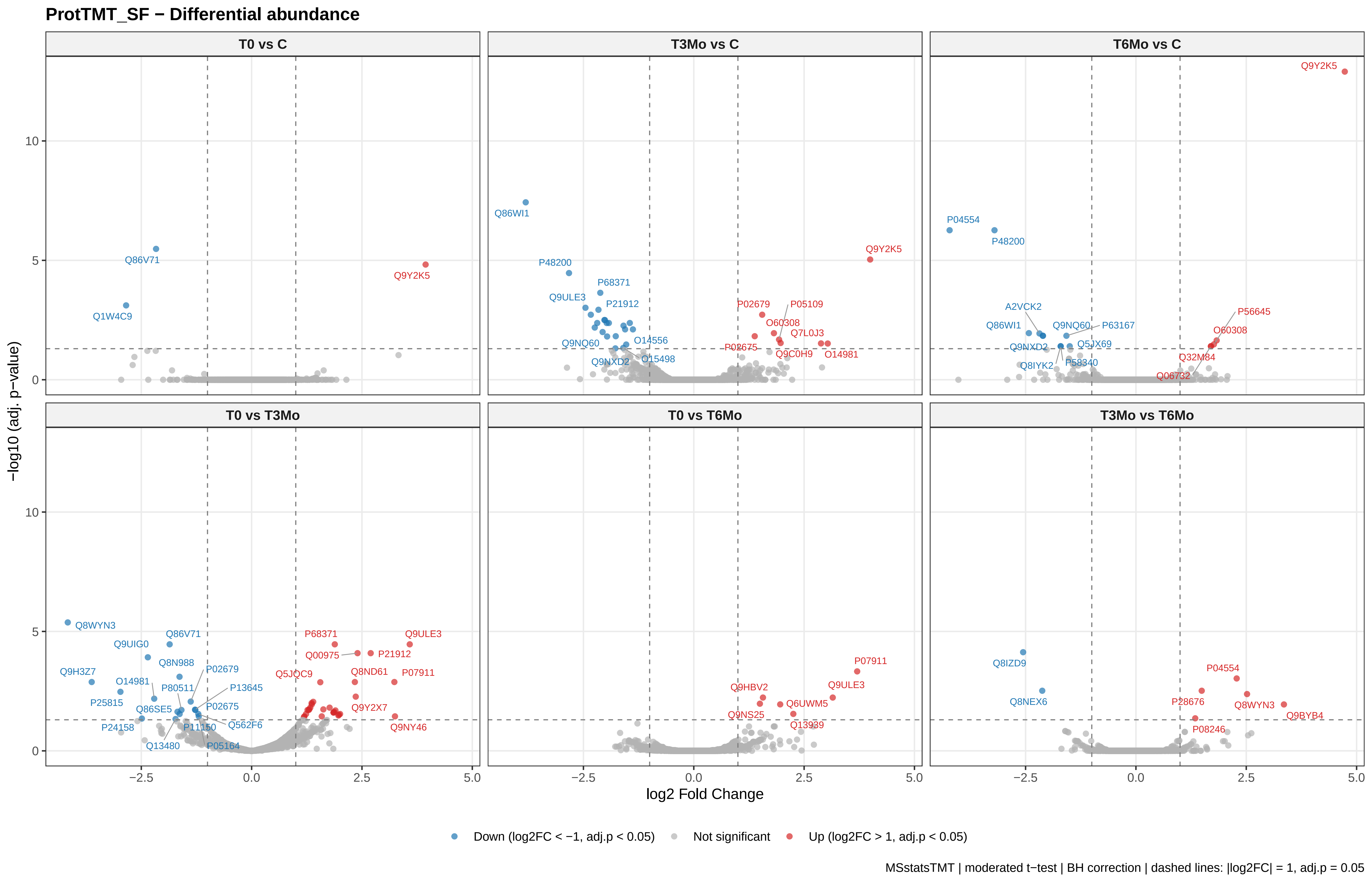
ProtTMT_SF − Differential abundance
T0 vs C
T3Mo vs C
T6Mo vs C
T0 vs T3Mo
T0 vs T6Mo
T3Mo vs T6Mo
Q86V71
Q1W4C9
Q9Y2K5
Q86WI1
P48200
P68371
Q9ULE3
P21912
Q9NQ60
O14556
Q9NXD2
O15498
P02679
P05109
O60308
Q7L0J3
P02675
Q9C0H9
O14981
P04554
A2VCK2
P56645
Q86WI1
Q9NQ60
P63167
Q9NXD2
Q5JX69
Q8IYK2
P58340
Q32M84
Q06732
Q8WYN3
Q9UIG0
Q86V71
Q8N988
Q9H3Z7
O14981
P25815
P80511
P02679
P13645
Q86SE5
P02675
P24158
P11150
Q562F6
Q13480
P05164
P68371
Q00975
P21912
Q9ULE3
Q5JQC9
Q8ND61
P07911
Q9Y2X7
Q9NY46
Q9HBV2
Q6UWM5
Q9NS25
Q13939
Q8IZD9
Q8NEX6
P28676
P08246
Q8WYN3
Q9BYB4
−log10 (adj. p−value)
log2 Fold Change
−2.5
0.0
2.5
5.0
0
5
10
Down (log2FC < −1, adj.p < 0.05)
Not significant
Up (log2FC > 1, adj.p < 0.05)
MSstatsTMT | moderated t−test | BH correction | dashed lines: |log2FC| = 1, adj.p = 0.05
