# Supplementary material for: The impact of significant weight loss following bariatric surgery on the differential expression of phosphopeptides in human seminal fluid
Source: Front Endocrinol (Lausanne). 2026 Oct 7;17:1907857. doi: 10.3389/fendo.2026.1907857 (PMC13684861; doi:10.3389/fendo.2026.1907857)
Supplement: Supplementary file 5 [file DataSheet5.pdf]

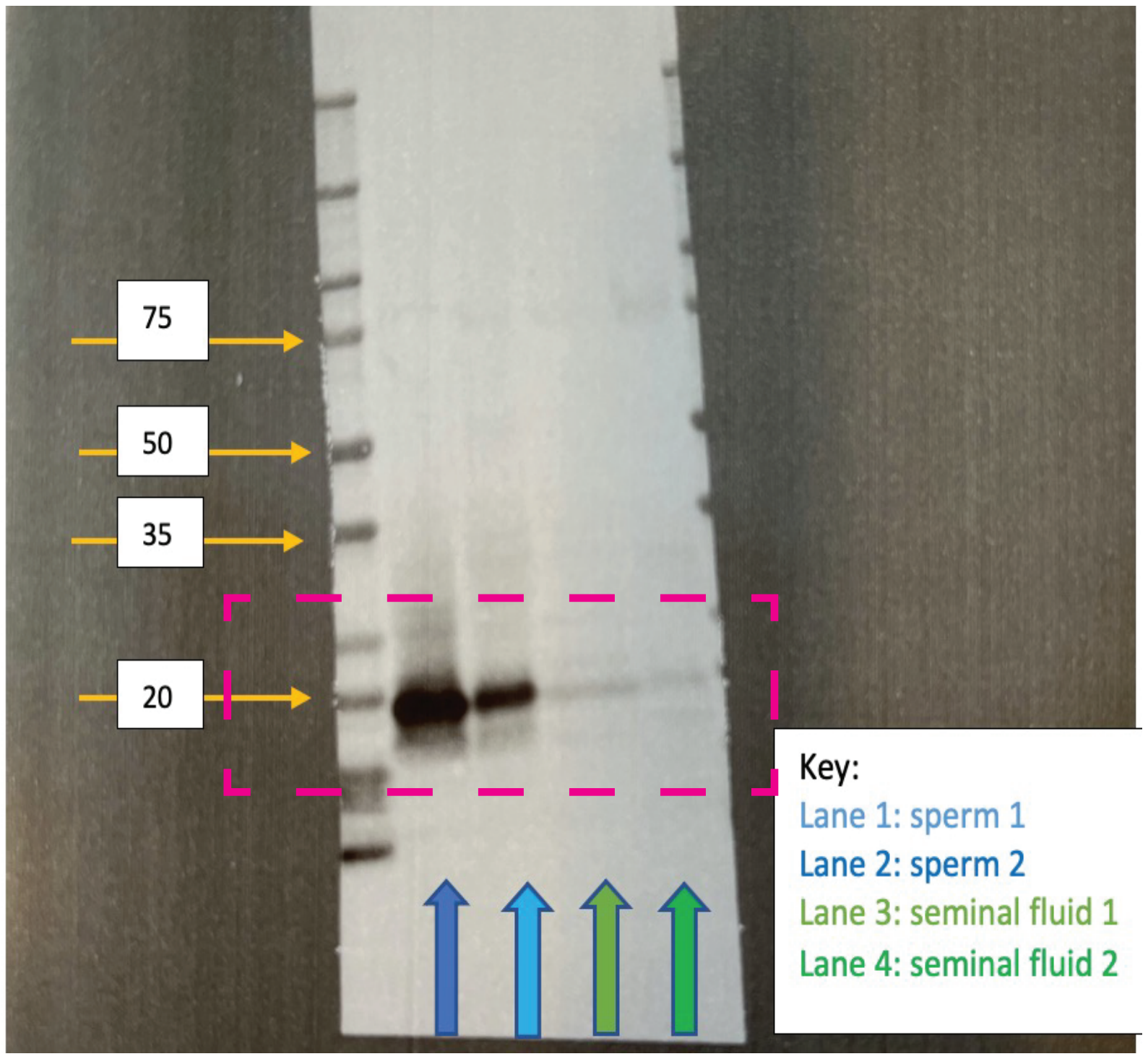

PMR2 antibody (1:1000 dilution) in 4-15% BIORAD precast gel; load 30ug (15ug @ 2ug/uL)
MW PMR2: 13,501

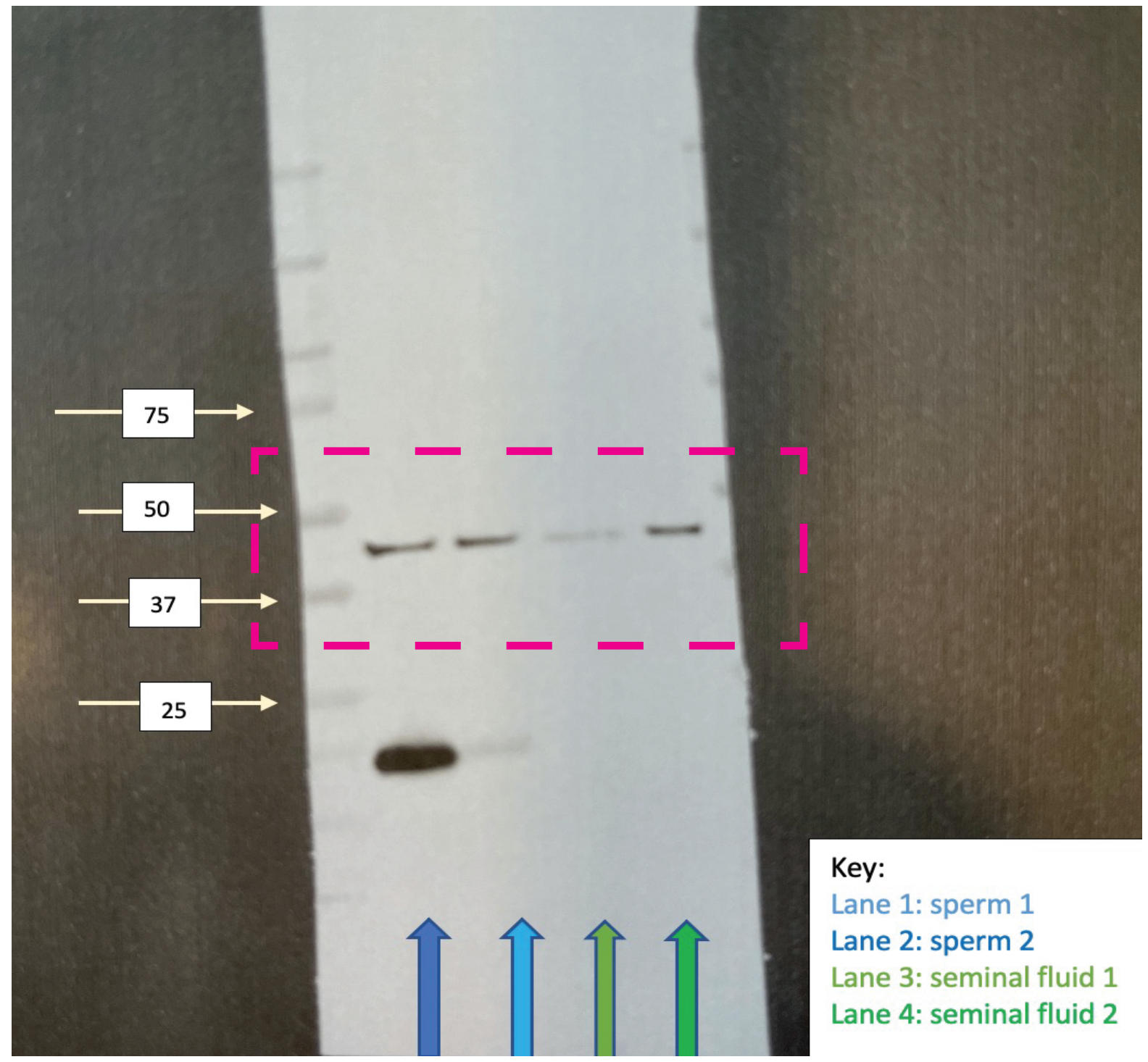

PMR2 antibody stripped, protein quantification performed with ACTIN antibody
MW: 41,373

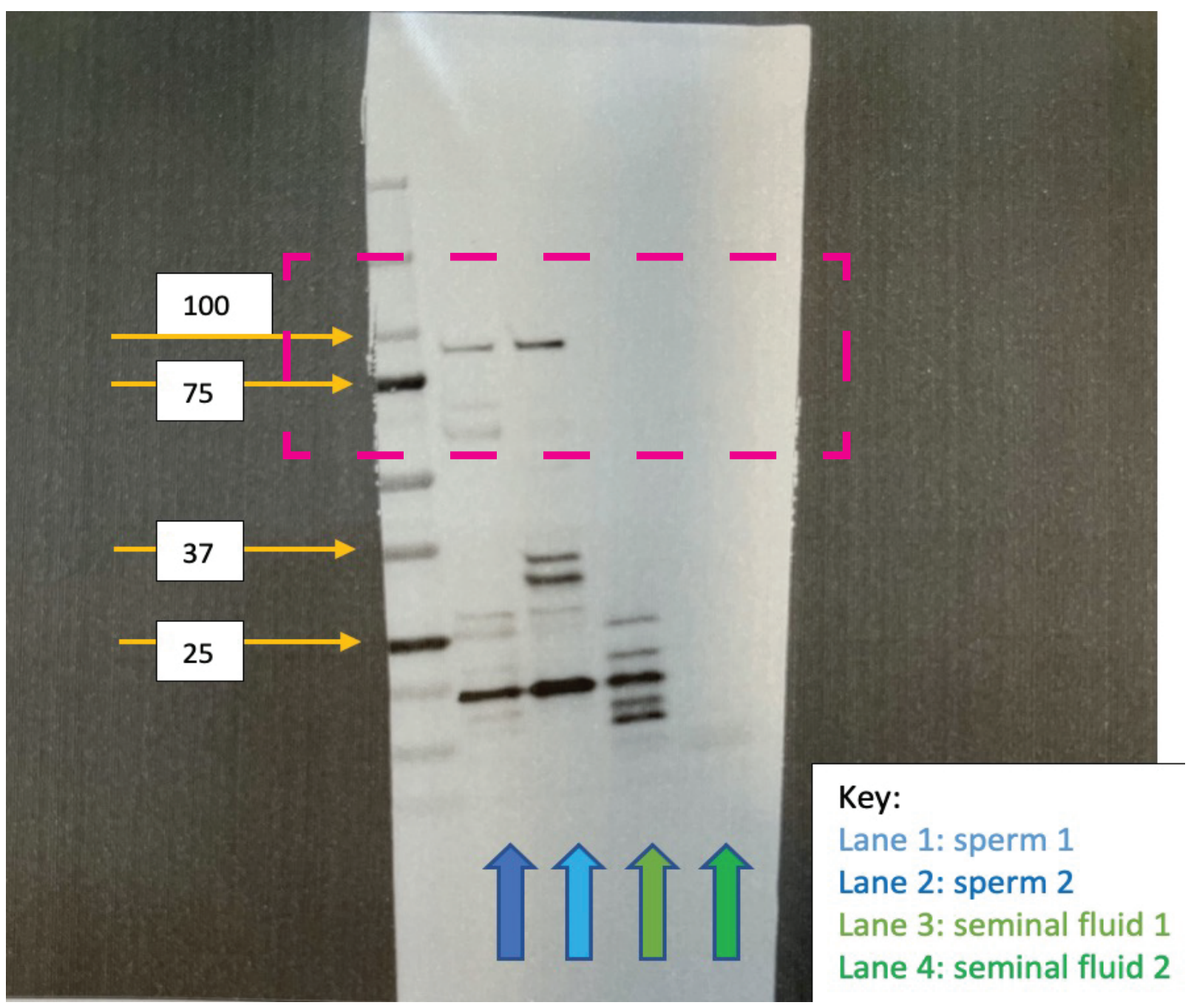

ADAM2 antibody (1:1000 dilution) in 4-15% BIORAD precast gel; load 30ug (15ug @ 2ug/uL)
MW ADAM2: 82, 457
(date of gel 6/10/22)

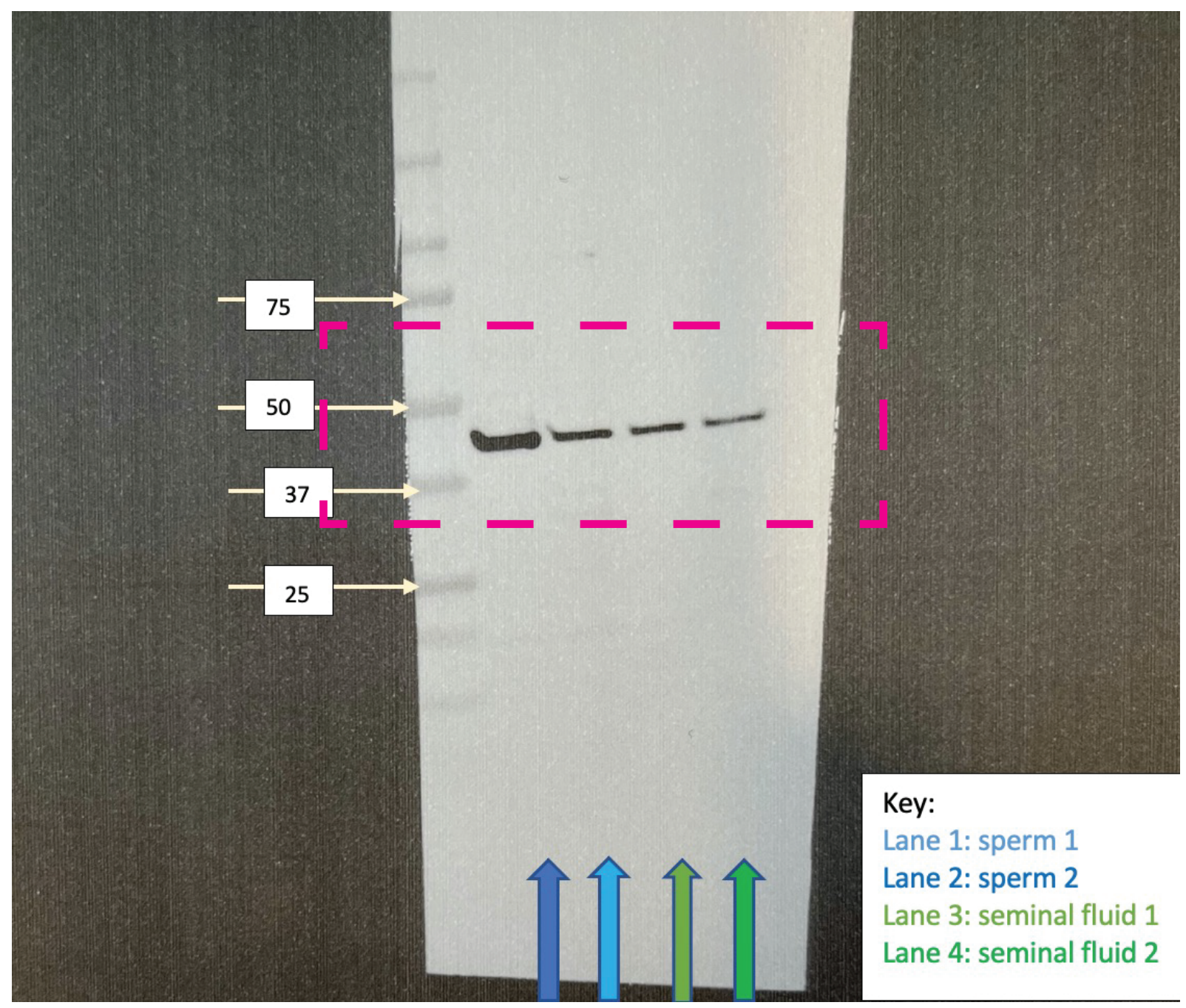

ADAM2 antibody stripped, protein quantification performed with ACTIN antibody
MW: 41,373
